# Method to estimate the basal turn length in inner ear malformation types

**DOI:** 10.1038/s41598-022-23911-5

**Published:** 2023-01-05

**Authors:** Afrah Alshalan, Fida Almuhawas, Salman Alhabib, Nezar Hamed, Yassin Abdelsamad, Anandhan Dhanasingh

**Affiliations:** 1 King Abdullah Ear Specialist Center (KAESC), College of Medicine, King Saud University, PO Box 72418, Riyadh, 23235 Saudi Arabia; 2 Department of Otolaryngology-Head and Neck Surgery, College of Medicine, Jouf university, Skaka, Saudi Arabia; 3 Research Department, MED-EL GmbH, Riyadh, Saudi Arabia; 4 Research and Development, MED-EL, Innsbruck, Austria; 5 Department of Translational Neurosciences, Faculty of Medicine and Health Sciences, University of Antwerp, Antwerp, Belgium

**Keywords:** Cochlea, Inner ear

## Abstract

The mathematical equations to estimate cochlear duct length (CDL) using cochlear parameters such as basal turn diameter (A-value) and width (B-value) are currently applied for cochleae with two and a half turns of normal development. Most of the inner ear malformation (IEM) types have either  less than two and a half cochlear turns or have a cystic apex, making the current available CDL equations unsuitable for cochleae with abnormal anatomies. Therefore, this study aimed to estimate the basal turn length (BTL) from the cochlear parameters of different anatomical types, including normal anatomy; enlarged vestibular aqueduct; incomplete partition types I, II, and III; and cochlear hypoplasia. The lateral wall was manually tracked for 360° of the angular depth, along with the A and B values in the oblique coronal view for all anatomical types. A strong positive linear correlation was observed between BTL and the A- (r^2^ = 0.74) and B-values (r^2^ = 0.84). The multiple linear regression model to predict the BTL from the A-and B-values resulted in the following equation (estimated BTL = [A × 1.04] + [B × 1.89] − 0.92). The manually measured and estimated BTL differed by 1.12%. The proposed equation could be beneficial in adequately selecting an electrode that covers the basal turn in deformed cochleae.

## Introduction

The clinical implication of estimating the cochlear duct length (CDL) is based on the selection of cochlear implant (CI) electrode array length. Estimation of CDL in a normally developed cochlea with two and a half turns, based on a single linear measurement (A-value), was first proposed by Escude et al.^1^. A mathematical equation was formulated to estimate the CDL for various angular insertion depths, from 360° to a maximum of 900°, along the lateral wall (LW). Since then, several studies have reported the fine-tuning of Escude’s CDL equation to make it applicable in the selection of the CI electrode array length and mapping the entire frequency range of the cochlea^2–4^. To bring these applications to clinical use, otological pre-planning software tools such as OTOPLAN^5^, which is clinically accepted, and NAUTILUS^6^, which is in the research phase, have been developed by different organizations. These clinical pre-planning tools are the result of several years of research efforts involving clinical and micro (µ)-computed tomography (CT) images of the normal anatomy of the cochlea developed with two and a half turns.

Anatomical abnormalities of the inner ear range in severity in 20–25% of children with congenital hearing loss^7^. Enlarged vestibular aqueduct syndrome (EVAS) is the mildest form of malformation, with almost a regular cochlear anatomy but with an enlarged vestibular sac. Incomplete partition (IP) type II, also called Mondini’s deformity, involves a better development of the basal turn, leaving the middle and apical turns of the cochlea cystic. IP type I is more severe in terms of anatomical deformation, with the cochlear portion being completely cystic and separated from the dilated vestibule, while IP Type III is more severe in terms of anatomical deformation with the cochlear portion completely lacking the central modiolus trunk, inter-scalar separation, and wide internal auditory canal directing opening up to the basal turn^8^. Measurement of the A-value is feasible in the majority of IEM types^9^. However, mathematical equations to estimate the CDL have not been formulated for cochleae with the IEM types.

It is commonly considered safe to place an electrode array to cover the first 360° of the angular depth in IP types I and III because of the cystic nature of the cochlea, with no clear distinction between scalar compartments^10,11^. More electrical coverage can be provided in IP II and EVAS types depending on how far the cochlear lumen is further developed before the cystic apex and the number of turns available in the cochlear hypoplasia (CH) type with a clinical implication of selecting an appropriate electrode array length. This raised our interest in formulating a mathematical equation to estimate the BTL in IEM types by applying the basal turn diameter (A value) and width (B value), as described by Escude et al.^1^.

## Methods

### Image analyses

**Figure 1:**
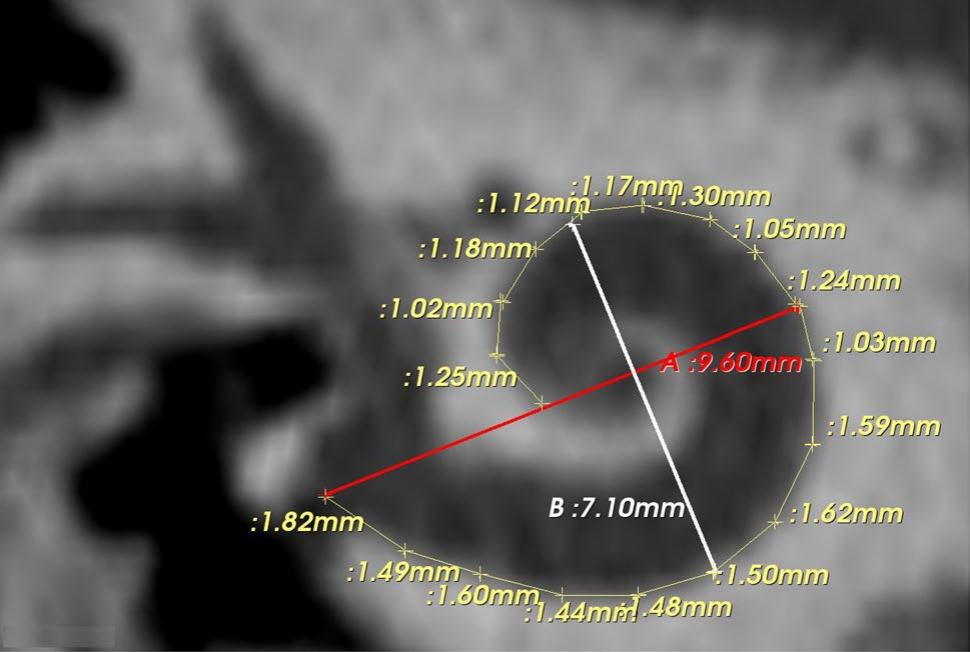
Oblique coronal view/cochlear view showing the A- (red line), and B- (white line) values and the lateral wall length measurements.

The CT scans from 2013 until the end of 2021 were considered for analysis. Based on the radiologist's report, CT scans with cochlear aplasia, ossified cochleae, temporal bone fracture, and common cavity were excluded. Inclusion criteria included CT scans with other cochlear malformation types, including normal anatomy (NA) in any age group and gender. The CT images were analyzed using 3D slicer software, version 4.10.2, freeware (https://www.slicer.org/). The A-value was measured in cochlear view starting at the entrance of the round window (RW) and passing through the mid-modiolar section to the opposite side of the LW in “cochlear view” as described previously by Escude et al.^1^. The B value was measured perpendicular to the A value in the cochlear view, which measured the longest distance. The BTL was tracked manually using small segments following the LW in the oblique coronal view of the cochlear basal turn from the RW to an angular depth of 360°. Figure 1 shows A- and B-values, and BTL manually measured in the cochlear view.

### Statistical analyses

The A- and B-values and the BTL of cochleae with NA and IEM types were compared using two-sample t-tests with unequal variance in Microsoft Excel for Office 356 (version 2020). Regression estimates between A versus B-values, A versus BTL, and B versus BTL were determined using the data analysis tool in Microsoft Excel. A multiple linear regression model was used to formulate a mathematical equation to estimate the cochlear BTL from the A and B values as inputs. The p-value was used to test the null hypothesis and determine whether it was accepted. Statistical significance was considered at a p-value < 0.05, with a confidence interval of 95%. The strength of the association between a single dependent and several independent variables was evaluated using multiple regression. In the current study, we used multiple regression analysis to formulate a mathematical equation to estimate cochlear BTL using the A and B values as inputs. The relative error (%) between the measured and estimated BTL was calculated by dividing the difference between the measured (M) and estimated (E) BTL values by the measured BTL value.

$${\text{The}}\,{\text{relative}}\,{\text{error}}\,\left( \% \right) \, = \, \left( {\left[ {{\text{M}} - {\text{E}}} \right]/{\text{M}}} \right) \, \times { 1}00.$$

M represents the measured BTL, and E is the estimated BTL.

### Ethics declarations

The study was conducted in accordance with the Declaration of Helsinki and approved by the local institutional review board of King Saud University (No. 20/0091/IRB).

### Informed consent

The informed consent was waived off due to retrospective nature of the study by the same local committee.

## Results

### Data analyses

**Figure 2:**
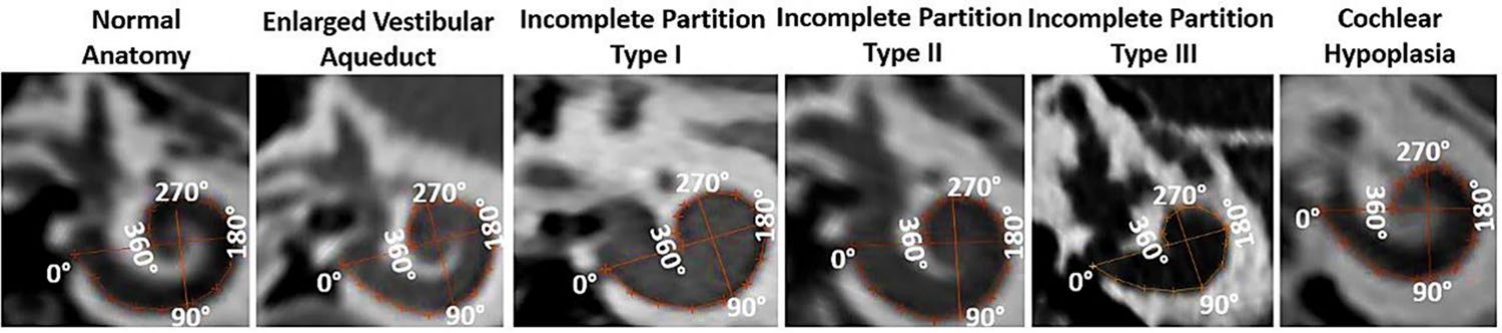
Oblique coronal views showing the cochlear basal turn of various inner ear anatomical types displaying the A- (long axis line), B- (short axis line) value, and basal turn length (BTL) manually tracked (360° coverage).

Of the 95 CT scans investigated, IEM types including EVAS with better cochlear formation were identified in 24, IP type I in 22, IP type II with EVA in 12, IP type III in 12, and CH type in 14 CT scans. The CT scans with NA type from the last 11 consecutive CI surgeries from our center were used as controls to compare the cochlear dimensions between the IEM and NA types. Within the CH type, the basal turn appeared normal, and with further development of the cochlear turns in five CT scans, leaving the other nine CT scans only with the basal turn. Figure 2 captures the inner ear of different anatomical types oriented in the oblique coronal plane showing the A- and B-values, and BTL. The insertion depth in angulation starts at 0° at the RW level, the intersection of the A- and B-value lines separates each quadrant, and each quadrant is counted in 90° increments.

**Figure 3:**
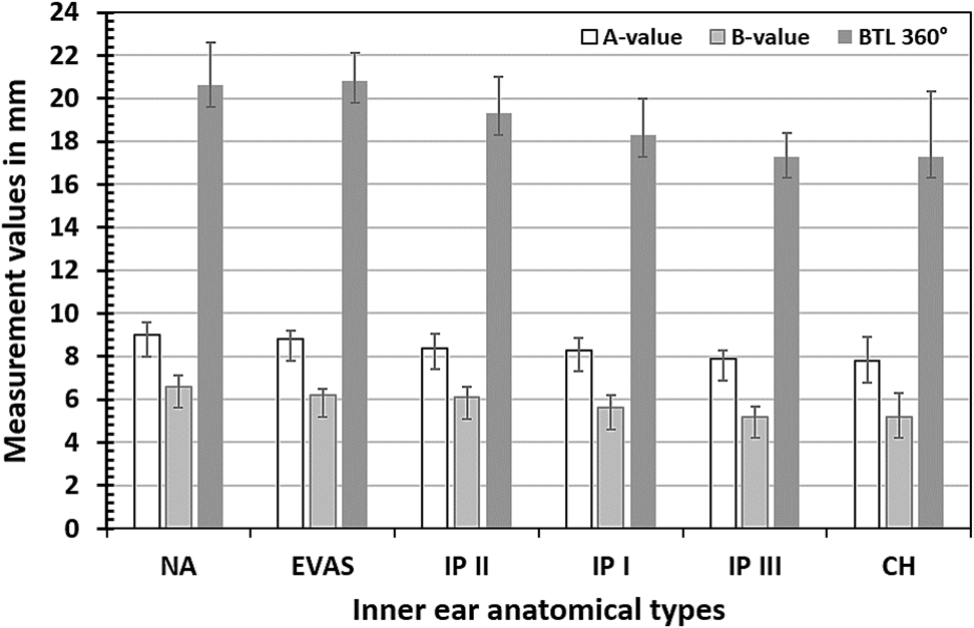
The mean and standard deviation of A-, B-value, and measured basal turn length (BTL) (360° coverage) for various inner ear anatomical types.

Table 1 displays the measured A- and B-values from all the samples taken for analysis. The A- and B-values (mean ± standard deviation) for each group are shown in Fig. 3. The A-values of the NA compared to those of the cochleae with IP types I (*p* = 0.003), II (*p* = 0.02), III (*p* < 0.001), and CH (*p* < 0.01) were significantly different. IP types I (*p* = 0.01) and II (*p* = 0.03) were also significantly different from IP type III in terms of A-value. IP type III was not significantly different from CH type (*p* = 0.36). The B-values of NA compared to the cochleae with IP types I (*P* < 0.001), II (*P* = 0.03), III (*P* < 0.001), and CH (*P* < 0.0001) were significantly different. IP type I (*p* = 0.1) and CH (*p* = 0.4) were not significantly different from IP type III in terms of B-value. The BTL of the NA cochleae was significantly different from that of the cochleae with IP types I (p < 0.01), II (p < 0.05), III (p < 0.001), and CH (p < 0.01). The BTL of IP type I was not significantly different from that of IP type II (*P* = 0.054). The BTL of IP type III was significantly different from that of IP type II (*p* < 0.01), but not with IP type I (*p* = 0.054).

**Table 1:** A- and B-values measured from all the anatomical types.

Types	NA		EVAS		IP I		IP II		IP III		CH	
Parameter	A (mm)	B (mm)	A (mm)	B (mm)	A (mm)	B (mm)	A (mm)	B (mm)	A (mm)	B (mm)	A (mm)	B (mm)
1	8.95	6.48	8.30	6.20	8.81	6.03	7.61	5.89	7.80	4.72	9.37	6.93
2	8.98	6.02	9.30	6.55	8.63	6.23	8.00	5.93	8.44	5.38	6.70	4,50
3	8.10	5.80	9.30	6.30	9.06	6.13	8.96	6.02	8.25	5.56	7.20	4.40
4	8.78	6.41	9.12	6.10	8.12	5.67	9.71	7.04	7.53	4.54	7.30	5.20
5	8.84	6.06	9.50	6.67	7.79	5.15	8.00	6.98	7.70	5.03	9.00	6.30
6	10.1	7.72	8.80	6.30	8.37	5.59	8.40	6.03	7.45	6.07	9.30	6.70
7	8.14	6.55	8.22	6.20	8.19	6.15	7.70	6.21	8.53	5.73	7.10	4.50
8	8.70	6.49	8.50	6.40	7.71	4.91	8.30	6.05	7.86	5.34	7.80	4.90
9	9.32	6.75	8.99	6.55	7.96	5.37	8.00	6.19	7.81	5.27	6.60	3.20
10	9.27	6.84	8.76	6.30	8.11	5.03	8.70	6.49	7.74	5.03	6.60	4.50
11	9.60	7.10	9.00	6.30	8.60	5.26	9.40	5.58	7.61	5.94	7.70	6.00
12	–	–	8.50	6.10	8.40	5.89	8.00	5.60	8.14	5.17	8.80	6.40
13	–	–	8.90	6.30	8.50	5.67	–	–	–	–	6.90	4.10
14	–	–	9.20	5.70	9.46	5.56	–	–	–	–	9.30	4.70
15	–	–	9.20	6.00	7.27	4.53	–	–	–	–	–	–
16	–	–	9.10	6.40	8.00	5.65	–	–	–	–	–	–
17	–	–	9.0	6.60	8.40	6.46	–	–	–	–	–	–
18	–	–	8.50	5.90	8.71	6.00	–	–	–	–	–	–
19	–	–	8.30	6.21	8.30	5.88	–	–	–	–	–	–
20	–	–	8.55	6.05	7.25	4.29	–	–	–	–	–	–
21	–	–	8.55	6.20	8.98	5.67	–	–	–	–	–	–
22	–	–	8.30	5.30	8.53	6.42	–	–	–	–	–	–
23	–	–	9.00	6.20	–	–	–	–	–	–	–	–
24	–	–	8.70	5.30	–	–	–	–	–	–	–	–
Mean ± Std. dev	8.98 ± 0.58	6.56 ± 0.53	8.82 ± 0.37	6.17 ± 0.34	8.32 ± 0.54	5.61 ± 0.57	8.39 ± 0.66	6.16 ± 0.46	7.90 ± 0.35	5.31 ± 0.46	7.83 ± 1.08	5.16 ± 1.11
Range	8.10–10.10	5.80–7.71	8.22–9.50	5.70–6.67	7.25–9.46	4.29–6.46	7.61–9.71	5.58–7.04	7.45–8.53	4.54–6.07	6.60–9.35	3.20–6.93

*NA* normal anatomy, *EVAS* enlarged vestibular aqueduct syndrome, *IP* incomplete partition type 1, *IP II* incomplete partition type 2, *IP III* incomplete partition type 3, *CH* cochlear hypoplasia.

**Figure 4:**
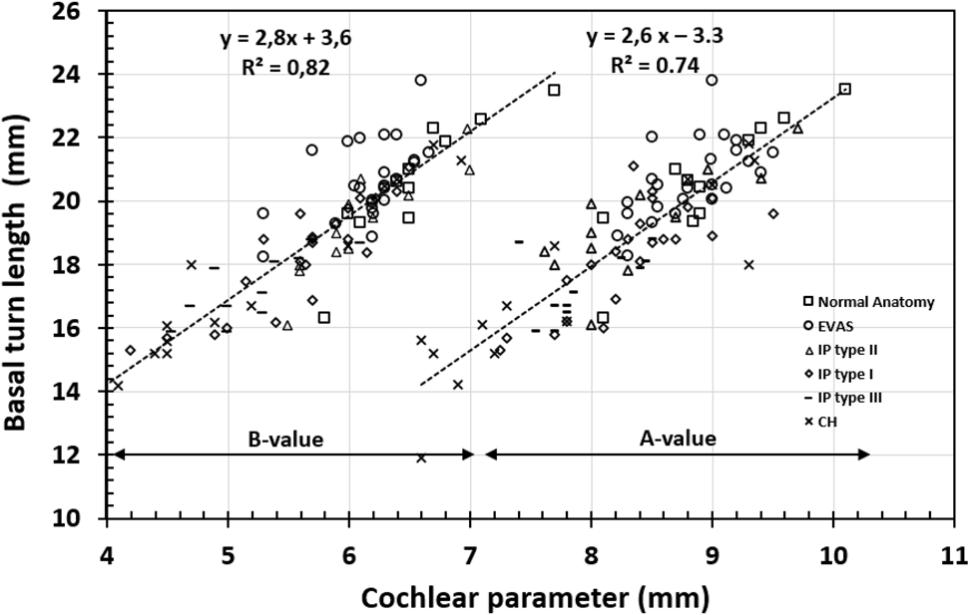
Correlation between basal turn length (BTL) and A-, B-values of all the anatomical types taken for analysis.

Both A- and B-values showed a strong positive linear correlation with the BTL (r^2^ of 0.8 and 0.74, respectively) when the data points of all anatomical types were collectively taken for analysis, as shown in Fig. 4.

### Prediction of BTL along the LW from basic cochlear parameters

The multiple linear regression model to predict the BTL from the A- and B-values resulted in the following equation (Eq. 1) with r^2^ = 0.9. The A- and B-values were measured in millimeters.

1 $${\text{Estimated}}\,{\text{BTL}} = A \times 1.04 + B \times 1.89 - 0.92.$$

Table 2 displays the BTL values measured manually and those estimated using Eq. (1).

**Table 2:** Basal turn length (BTL) (mm) as measured and estimated, along with the relative error between the two values in percentage.

Types	NA			EVAS			IP I			IP II			IP III			CH		
No.	M	E	Err. %	M	E	Err. %	M	E	Err. %	M	E	Err. %	M	E	Err. %	M	E	Err. %
1	20.43	20.64	0.9	19.95	19.43	2.6	19.80	19.64	0.8	18.40	18.13	1.5	16.71	16.11	3.7	21.29	21.90	2.7
2	19.61	19.80	0.9	21.23	21.13	0.4	20.10	19.83	1.3	18.50	18.61	0.5	18.09	18.03	0.3	15.20	14.55	4.4
3	19.30	18.47	4.5	20.90	20.76	0.6	20.10	20.09	0.0	21.00	19.78	6.1	18.26	18.17	0.5	15.20	14.88	2.1
4	20.64	20.33	1.5	20.41	20.09	1.5	16.90	18.24	7.3	22.30	22.48	0.8	15.91	15.50	2.7	16.70	16.50	1.2
5	19.34	19.73	1.9	21.54	21.57	0.1	17.47	16.92	3.2	19.90	20.59	3.3	15.85	16.60	4.4	20.50	20.35	0.7
6	23.51	24.27	2.7	20.42	20.14	1.4	18.10	18.35	1.3	20.20	19.21	5.1	18.71	18.30	2.2	21.80	21.42	1.8
7	19.47	19.93	2.3	18.90	19.35	2.3	18.40	19.22	4.2	18.08	18.82	4.3	18.81	18.78	0.1	16.10	14.97	7.5
8	21.00	20.39	2.9	20.70	20.02	3.4	15.80	16.38	3.5	17.80	19.15	7.0	17.12	17.35	1.3	16.20	16.45	1.5
9	22.27	21.53	3.4	21.30	20.81	2.3	16.20	17.51	7.4	16.10	19.10	15	16.50	17.16	3.8	11.90	11.99	0.7
10	21.90	21.65	1.1	20.05	20.10	0.2	16.00	17.02	6.0	19.48	20.39	4.8	16.68	16.64	0.3	15.60	14.45	7.9
11	22.60	22.48	0.5	20.50	20.35	0.7	18.84	17.97	4.8	20.73	19.40	6.8	18.22	18.22	0.0	18.60	18.43	0.9
12	–	–	–	22.00	19.45	13.1	19.26	18.95	1.6	18.96	17.98	5.6	17.30	17.32	0.1	20.70	20.33	1.8
13	–	–	–	22.10	20.24	0.1	18.73	18.64	0.5	–	–	–	–	–	–	14.20	14.01	1.3
14	–	–	–	21.60	19.42	9.1	19.59	19.43	0.8	–	–	–	–	–	–	18.00	17.64	2.0
15	–	–	–	21.90	19.99	11.2	15.69	15.20	3.2	–	–	–	–	–	–	–	–	–
16	–	–	–	22.10	20.64	9.5	18.00	18.08	0.4	–	–	–	–	–	–	–	–	–
17	–	–	–	23.80	20.91	7.0	21.10	20.03	5.3	–	–	–	–	–	–	–	–	–
18	–	–	–	19.31	19.07	13.8	18.75	19.48	3.7	–	–	–	–	–	–	–	–	–
19	–	–	–	19.60	19.45	1.2	18.84	18.83	0.0	–	–	–	–	–	–	–	–	–
20	-	–	–	20.50	19.41	0.7	15.27	14.73	3.6	–	–	–	–	–	–	–	-	–
21	–	–	–	19.80	19.69	5.6	18.90	19.14	1.2	–	–	–	–	–	–	–	-	–
22	–	–	–	18.26	17.73	3.0	20.30	20.09	1.0	–	–	–	–	–	–	–	–	–
23	–	–	–	20.05	20.16	0.5	–	–	–	–	–	–	–	–	–	–	–	–
24	–	–	–	19.60	18.50	8.0	–	–	–	–	–	–	–	–	–	–	–	–
Range	19.3 –23.5	18.5 –24.2	0.52 –4.5	18.26 –23.8	17.7 –21.6	0.12 –13.8	15.3 –21.1	14.7 –20.1	0.2 –7.7	16.1 –22.3	17.9 –22.4	0.0 –15.7	15.8 –18.8	15.5 –18.8	0.0 –4.5	11.9 –21.8	11.9 –21.9	0.75 –7.97
Mean ± Std. dev	20.9 ± 1.5	20.8 ± 1.5	2.1 ± 1.3	20.6 ± 1.2	19.9 ± 0.88	4.1 ± 0.12	18.3 ± 1.7	18.4 ± 1.5	2.9 ± 2.3	19.3 ± 1.6	19.4 ± 1.3	5.1 ± 4.0	17.3 ± 1.1	17.3 ± 1.1	1.6 ± 1.6	17.2 ± 2.9	16.9 ± 3.0	2.66 ± 2.3

*NA* normal anatomy, *EVAS* enlarged vestibular aqueduct syndrome, *IP* incomplete partition type 1, *IP II* incomplete partition type 2, *IP III* incomplete partition type 3, *CH* cochlear hypoplasia, *M* measured BTL, *E* estimated BTL, *Err* relative error.

Earlier in 2006, Escude et al. formulated a mathematical equation to estimate the CDL along the LW, using the A-value alone as input, and their mathematical equation could be adapted to estimate the BTL by changing the value of Ө to 360°.

$${\text{BTL}} = { 2}.{62 } \times {\text{ A }} \times {\text{ ln }}\left( {{1} + \, \left[ {{36}0^\circ /{235}} \right]} \right).$$

Escude’s estimation of BTL was on average 1.03 mm for NA, 1.55 mm for EVAS, and 1.91 mm, 0.97 mm, and 1.89 mm for IP types I, II, and III, respectively higher in comparison to our estimation.

## Discussion

Estimation of the CDL using mathematical equations for various angular insertion depths by applying the A- and/or B-values as the input has been validated for NA cochleae^2–4^. These mathematical equations have been included in clinically approved otological pre-planning software tools for estimating the cochlear size and choosing appropriate electrode lengths^5^. However, the IEM types are still not completely addressed when estimating cochlear length. Therefore, this study attempted to find a clinically feasible solution to estimate the BTL in IEM types with A and B values as input.

The LW can be manually tracked/measured for an angular depth of 360° from clinical CT images in the oblique coronal view, and this has been reported in NA cochleae by Adunka et al.^12^. This motivated us to manually measure the BTL (360°) following the LW from 95 datasets, including IEM types of EVAS; IP types I, II, III; and CH, and compare it with the NA cochleae. While it is feasible to measure the BTL manually from clinical CT, the BTL can be estimated from cochlear parameters such as A- and B-values.

Plotting the A and B values against the BTLs of all anatomical types showed a strong positive linear correlation with the overlapping of data points from all six anatomical types studied. This implies that the BTL (360° coverage) of all these anatomical types can be estimated from basic cochlear parameters, which is a novel finding of this study. From our clinical experience, we learned that placing a longer length electrode array (31 mm and 28 mm) beyond 360° of angular depth in IP type I resulted in tip fold-over, as the cochlear apex in IP type I beyond the 360° mark is cystic^13^. This was another reason that motivated us to look for a method to estimate BTL to limit electrode placement to 360° of angular depth in difficult anatomies.

In comparison to Escude’s mathematical equation involving the A-value alone as input, our estimation of BTL involves both A- and B-values as inputs, minimizing the error between the measured and estimated BTLs. Khurayzi et al.^14^ reported the importance of the B-value along with the A-value in defining the shape of the cochlear basal turn, which was reflected in minimizing the error in our study.

Considering only the eight CT image datasets that were used in the manual measurement of BTL in NA cochleae by Adunka et al., the number of CT image datasets used in the current study was relatively high. This increases the confidence level of the mathematical equation formulated for the estimation of BTL. The LW was observed extending beyond 360° in EVAS, IP type II, and in some samples of CH, which needs to be studied systematically in the future.

## Conclusion

The LW of IP types I and III can be tracked consistently to 360° of angular depth in the cochlear view, whereas it extends more than 360° in IP type II, EVAS, and CH types. The finding of a positive linear correlation between BTL and cochlear parameters supports the estimation of BTL using the basic cochlear parameters of A and B values in the IEM types. The mathematical equation proposed in this study could be helpful in choosing electrode array length covering the basal turn in malformed cochleae.
